# Copayment and recommended strategies to mitigate its impacts on access to emergency medical services under universal health coverage: a case study from Thailand

**DOI:** 10.1186/s12913-016-1847-y

**Published:** 2016-10-21

**Authors:** Paibul Suriyawongpaisal, Wichai Aekplakorn, Samrit Srithamrongsawat, Chaisit Srithongchai, Orawan Prasitsiriphon, Rassamee Tansirisithikul

**Affiliations:** Department of Community Medicine, Faculty of Medicine Ramathibodi hospital, Mahidol University, Bangkok, Thailand

**Keywords:** Emergency medical services, Copayment, Universal Health Coverage Schemes

## Abstract

**Background:**

Although bodies of evidence on copayment effects on access to care and quality of care in general have not been conclusive, allowing copayment in the case of emergency medical conditions might pose a high risk of delayed treatment leading to avoidable disability or death.

**Methods:**

Using mixed-methods approach to draw evidence from multiple sources (over 40,000 records of administrative dataset of Thai emergency medical services, in-depth interviews, telephone survey of users and documentary review), we are were able to shed light on the existence of copayment and its related factors in the Thai healthcare system despite the presence of universal health coverage since 2001.

**Results:**

The copayment poses a barrier of access to emergency care delivered by private hospitals despite the policy proclaiming free access and payment. The copayment differentially affects beneficiaries of the major 3 public-health insurance schemes hence inducing inequity of access.

**Conclusions:**

We have identified 6 drivers of the copayment i.e., 1) perceived under payment, 2) unclear operational definitions of emergency conditions or 3) lack of criteria to justify inter-hospital transfer after the first 72 h of admission, 4) limited understanding by the service users of the policy-directed benefits, 5) weak regulatory mechanism as indicated by lack of information systems to trace private provider’s practices, and 6) ineffective arrangements for inter-hospital transfer. With demand-side perspectives, we addressed the reasons for bypassing gatekeepers or assigned local hospitals. These are the perception of inferior quality of care and age-related tendency to use emergency department, which indicate a deficit in the current healthcare systems under universal health coverage. Finally, we have discussed strategies to address these potential drivers of copayment and needs for further studies.

**Electronic supplementary material:**

The online version of this article (doi:10.1186/s12913-016-1847-y) contains supplementary material, which is available to authorized users.

## Background

Many health authorities advocate for universal health coverage (UHC) regardless of economic status of a country citing potential benefits such as financial protection from catastrophic illnesses, increased access to healthcare, and reduction in out-of-pocket payments (OOP) [1, 2]. For decades, implementation of the UHC concept has not only been possible even in low income countries, but has also led to the expected benefits [3, 4]. To achieve UHC, at least three major functions need to pursue: raising revenues, pooling risk and purchasing health services [5]. Copayment is considered a means to reduce demand-side practices of over utilization of healthcare due to moral hazard or a form of revenue generation [6]. Regardless of its expected functions, copayment has been associated with dampening effect on demand for necessary services and can even lead to catastrophic spending [2, 7].

In 2001, a new major public-health financing program (the Universal Health Coverage Scheme, UCS) was introduced in Thailand providing coverage of healthcare extending to 75 % of the population. This major step advanced the national health insurance reforms towards full universal coverage on top of the previous public-health insurance schemes (Social Security Scheme, SSS and Civil Servant Benefit Scheme, CSMBS). Over a decade of the full universal coverage in Thailand, evidence has shown increased financial risk protection and increased service utilization [8]. Together it seems like copayment is not an issue of concern under the full universal coverage [9]. However, a national household survey in 2013 revealed that 39.2 % of UCS beneficiaries made use of outpatient care in public and private health facilities with OOP [10]. The figure for inpatient care with OOP was 8.5 %. It has been argued that the main reasons for the remaining OOP are beneficiaries bypassing the referral system or making use of private hospital inpatient care [11]. The prevalence of OOP charges may be caused by a range of factors including: absence of full population coverage; key services not being included in the benefit package; beneficiaries bypassing the assigned local providers or bypassing the referral system in accessing inpatient care; or inappropriate or illegal billing practices by regular providers [12].

In addition, users’ perception of quality of care can influence choices of providers hence resulting in OOP when bypassing the assigned local providers for provider of choice. Using multiple logistic regression model to analyse in-patient survey data, Cheng et al. revealed a strong association between perceived quality of care (technical capabilities or interpersonal skills) and choices of providers [13].

In April 2012, the Thai government undertook a nation-wide unified Emergency Medical Services (EMS) policy initiative in mobilizing private hospitals in addition to those participating in 2 major public health financing programs (SSS and UCS) to fill the perceived gap of access to EMS for all health insurance beneficiaries. The perceived gap of access was highlighted by the Prime Minister out of a concern of inequitable access due to different amount and mechanisms of payment among the three major schemes [14]. In other words, these private providers could be considered outside contracted private hospitals. As a result, the government established a specific purchasing model to compensate these private hospitals for provision of emergency department (ED) services and inpatient services. Given the compensation, policymakers expect that the service provision should be free from copayment. Apart from filling the gap, this policy also involved an implicit objective i.e., to make an attempt to unify purchasing models across the 3 schemes using the purchase of EMS as an entry point.

To guide the policy implementation, a two-phase independent evaluation was undertaken employing a mixed methods approach (to be presented in detail below) to shed light on the progression, obstacles and alternatives for policy adjustment. The first phase covered the first six months of the policy implementation from which findings and respective feedback to policymakers and stakeholders guided the second phase. An important finding from the first phase deals with patient outcomes. Using an administrative dataset of 22,900 records for the unified purchasing model, Paibul et al. reported a discrepancy in fatal and non-fatal outcomes at discharge from inpatient care among the beneficiaries of different schemes [15]. In comparison to the outcomes of CSMBS beneficiaries, UCS and SSS beneficiaries are 1.7 to 2.1 times more likely to be reported not-improved status or 1.2 to 1.9 times more likely to be reported dead upon discharge from inpatient care. The discrepancy was independent of age, sex, severity of medical conditions and length of stay according to multiple logistic regression analysis. The report indicated a possibility of the effect of differential affordability for copayment among different beneficiary groups contributing to disparity of the outcomes.

The copayment seems likely given the fact that there is no legal provision to support the policy expectation of no extra payment. In addition, the haste in policy implementation (less than 2 weeks in preparation phase) may preclude consultation with private providers about payment rates. Comparing the single payment rate for inpatient care to private providers of 10,500 baht per relative weight (RW) of diagnosis-related-group (DRG) under this policy to that of 15,000 baht per RW under an earlier program of the UCS, it could readily be argued that perceived under payment among the private providers could be a crucial issue. Following the implementation, we sought to document the prevalence of copayment under the new policy and to identify the main drivers of copayments and to evaluate the implications of copayments for access.

These are the key policy questions to be explored in this report. Answering these questions may be useful in the further development of laws, processes and institutional responsibilities that define private sector participation in the provision of essential health services under UHC. Notwithstanding careful searching of the main bibliographic databases covering health services no reports on the effects of copayment on utilization of EMS and factors related to the copayment in low and middle-income countries were identified.

### Settings

Over the last 3 decades, EMS systems in Thailand have gradually evolved in response to the emergency health needs. The EMS systems include pre-hospital ambulance services, medical emergency dispatch centers, ED services and inpatient definitive care organized mainly by public providers in rural and urban settings and to a certain extent by private providers in major cities.

Since 2001, UCS, SSS and CSMBS have mainly financed hospital emergency services including ED and inpatient definitive care. The payment methods and payment rates differ among the 3 schemes with some overlapping as follows.

Under the UCS, all the contracted hospitals, both public and private, are paid for EMS provided to a non-registered UCS member by fee for service (FFS) with a ceiling of 700 Baht for ED visits and DRG with global budget for inpatient care. FFS with ceiling was adopted to pay private hospitals outside the contract for both ED and inpatient care. Under the SSS, public hospitals are paid according to the billing for ED visits and the first 72 h of admission. For private non-registered hospitals, both under and outside the contract, the patients have to pay upfront and reimburse from the central fund by FFS with ceiling for both ED visits and the first 72 h of admission. After 72 h of admission, the registered hospital is fully responsible for the costs of service provided.

Under CSMBS payment methods, all public hospitals are reimbursed on a fee schedule for ED visits and on the DRG basis for inpatient care plus boarding and medical supply based on a predefined list. Prior to the policy initiative, the reimbursement for inpatient care to private hospitals was capped at 4,000 Baht (133 United States Dollar (USD)) per admission. There was no payment for ED service to private hospitals. Notably, a CSMBS beneficiary is not required to register to any hospitals, as opposed to those of SSS and UCS. Should there be a need for inter-hospital transfer after initial admission to a hospital for an emergency condition they are more likely to face difficulty in seeking another hospital for the transfer.

Without an exclusive dataset for ED services, an unofficial estimate of total ED visits to public hospital was reported to be 13 million annually [15]. Based on this figure and a reported figure of 32 % admission rate from ED [16], the annual workload of inpatient emergency cases for the whole country could be at least 416,000 per year. Despite the fact that private providers play a significant role in provision of emergency services in the country, there is no data on the service provision and expenditure [17].

### Policy interventions

The unified EMS policy aims to close the gap of access to EMS under the three health insurance schemes. The policy interventions include mobilizing private hospitals outside the previous contracts; unification of payment methods and payment rates for purchase of EMS from these private hospitals; public policy communication to inform the beneficiaries on the right of access without copayment; and monitoring of the progress for ongoing adjustment. The National Health Security Office (NHSO), in charge of UCS, is assigned to be the responsible agency in executing this policy. The unified purchasing model is characterized as single payment methods and single payment rates for ED visits and hospitalization to the outside contracted private hospitals. For ED visits, the payment is based on the fee schedule of the existing CSMBS protocol plus extra items beyond the fee schedule, but does not exceed 1,000 Baht. For hospitalization, DRG is a case-based payment at 10,500 Baht (approximately 350 USD) per adjusted RW which is used to reimburse the private hospitals. The private hospitals receive extra payments for high cost medications and medical instruments (e.g., prosthesis, intravascular stents). Financial audit of every claim is mandatory prior to the reimbursement. This includes checking severity classification, DRG related data, health insurance entitlement, patient identification (ID) code, hospital ID code, and date/time of hospital visit and discharge. NHSO also acts as the Clearing House to process reimbursement claim, patient complaints, and to coordinate, in collaboration with Ministry of Public Health (MOPH), inter-hospital transfer of patients. An administrative dataset was exclusively set up to facilitate the financial claim processes using online transactions.

As part of the policy implementation, the government made use of mass media and out-door media for public communication campaign to raise awareness of free access to all public and private hospitals nation-wide in case of a need for EMS despite the fact that supply-side interventions are limited to 255 outside contracted private hospitals (78.2 % of the total number of private hospitals). In addition to the unified payment mechanism, the MOPH held several official meetings with representatives of the private hospitals to press them to participate citing the mandate stipulated in The Sanatorium Act B.E. 2541. The law requires private hospitals to provide emergency care for any patients until the emergency condition has been stabilized without mentioning any hospital payments and definitions of the conditions. The combined supply-side and demand-side interventions of the programs mentioned are expected to work together in promoting better access to emergency services. It is noteworthy that the majority of the outside contracted private hospitals previously chose to focus on the high-end well-paid market of healthcare. Setting the payment rate without adequate participation of these private hospitals hence posted a challenge in their compliance to the service provision. The lack of any regulatory capacity to compel the private hospitals not to charge copayments above the EMS payments posted another challenge of copayment.

In practice, the program involved a large body of representatives mainly from the outside contracted private hospitals, 3 departmental units of the MOPH, and the 3 schemes. In every high-level decision-making forum, the Prime Minister chaired the meetings herself indicating a strong policy commitment. Monthly reports of the progress in policy implementation in terms of outputs and public responses (directly from the complaint channel and indirectly from mass media) were key inputs to the forums. Multiple stakeholders with multiple perspectives were brought into the policy arena along with the ambitious purpose of achieving more unified healthcare financing mechanism (after a decade of stagnation on this policy track) [8] and access to EMS expanded at the same time to support this policy initiative.

## Methods

This study was approved by the Institutional Ethical Review Board of the Faculty of Medicine, Ramathibodi Hospital.

Given the complicated financial arrangements and complexity of the policy implementation, we chose to use a mix-methods approach to fill the knowledge gaps. In principle, a mixed methods has been considered suitable for research questions fitting any of the following characteristics: exploring the meaning of a construct or phenomenon from more than one perspective; explanation of anomalous findings or getting behind the mechanism of action of an effect; theory development followed by testing/extension; measure development using grounded concepts; or augmenting evaluation studies with better understanding of intervention implementation [18–20].

In our case, the posted research questions seem to be, largely, in keeping with these characteristics. First, the complexity of the policy initiative precludes any single perspective to dominate making sense of the meanings of the policy phenomenon. Second, to understand the constraints and drivers regarding access to EMS and the underlying functions and mechanisms requires qualitative findings from in-depth interviews with multiple stakeholders and quantitative findings from the user complaint channel or more systematic user survey. The following describes details of the methods in the data acquisition.

It is notable that the two-phase evaluation was carried out with substantial participation from key actors from the inception (the evaluation planning) to drafting of the final reports. The key actors comprised the 3 schemes, MOPH as regulator, the Clearing House, and the Private Hospital Association. This participatory approach enabled the authors to get access to relevant policy documents and the administrative (electronic claim) dataset. Nonetheless, despite the involvement of the Private Hospital Association, the authors did not have access to any financial data from the targeted private hospitals. In addition, we could not access to the minutes of all the meetings chaired by the Prime Minister.

## Sources of information and data

### Primary data

#### In-depth interview

In order to explore potential explanatory factors of copayment, we employed in-depth interviews with key actors: regulators, private hospital directors, the Clearing House administrators and the administrators of the health insurance schemes (Table 1). Emerging themes identified from the in-depth interviews covered: operational definition of emergency conditions, mechanisms to ensure common understanding and acceptance of the definition among the key stakeholders, regulatory function and mechanism to enhance provider compliance to the laws and the policy intentions, provision of care within each hospital, and feedback on the payment and the regulation (Additional file 1).

**Table 1:** Summary of participants in in-depth interviews

Position	Region			
	Southern	Northeastern	Northern	Bangkok
Hospital directors	2	2	5	3
Financial staff	2	2	5	3
Nurses	5	2	8	6
Doctors	2	2	5	3
Administrators of health insurance schemes and the Clearing House	N.A.	N.A.	N.A.	6

*N.A*. not applicable

Selected private hospitals were purposively chosen aiming at hospitals with billing at the 3rd quartile (6,525 USD) or over and those under the 2nd quartile (3,597 USD) or below. This selection criterion enabled us to contrast factors, which might explain relevant hospital behaviors (service provision, pricing policies, and opinions about the policy initiative). Distribution of selected private hospitals and their locations is shown in Table 2. In each hospital, participants included a hospital director, ED staff (doctors and nurses), and a financial officer.

**Table 2:** Number of selected private hospitals by hospital location and amount of billing

Amount of billing	Hospital location			
	Northern	Southern	Northeastern	Bangkok
3rd quartile and above	2	1	1	2
2nd quartile and below	3	2	1	1

#### User survey

To ascertain the prevalence of copayment, independent of those from the official complaint dataset, a telephone survey was (Additional file 2) conducted on 128 randomly chosen patients from the total number of 1,745 patients in April 2012, which was the first month of the policy implementation. The survey explored the following issues: perception of the policy messages, access to care (mode of access, choices of hospitals, and reasons for the choice), opinions about the services and copayment.

### Secondary data

#### Emergency medical claim online (EMCO) data set

The EMCO dataset included 43,588 records of financial claims from 225 private hospitals or 88.2 % of the expected number (providing secondary and/or tertiary medical care with a total capacity of inpatient care of 29,734 beds or 20.6 % of the whole country figure) during April 2012 to December 2013. Ninety one percent of the records dealt with inpatient care. The EMCO dataset contains 50 variables including those extracted for the present study: amount of claims, amount of reimbursement, claim approval status, adjusted relative weight AdjRW), and service delivery data (severity classification, principal diagnosis, type of hospital visit (ED or inpatient department), date/time at discharge, and outcomes at discharge.

#### Documentary review

We reviewed documents to supplement and triangulate findings from the user survey and in-depth interviews. We searched documents purposively using Google Scholar and Google Search and PubMed based on the following keywords: EMS, regulation, definition, private hospital, payment methods, access, ED, outpatient, inpatient, annual report of regulatory agencies. We contact key actors mentioned above for relevant documents not accessible via internet. From the Clearing House, we try to identify 3 major types of documents i.e., a report of user complaints, a summary report on progress of the program and telephone survey reports. Finally, we employ snowball search strategy to obtain further documents to better understand emerging issues from review of previous documents and in-depth interview.

### Data analysis

#### Quantitative data

**Fig. 1:**
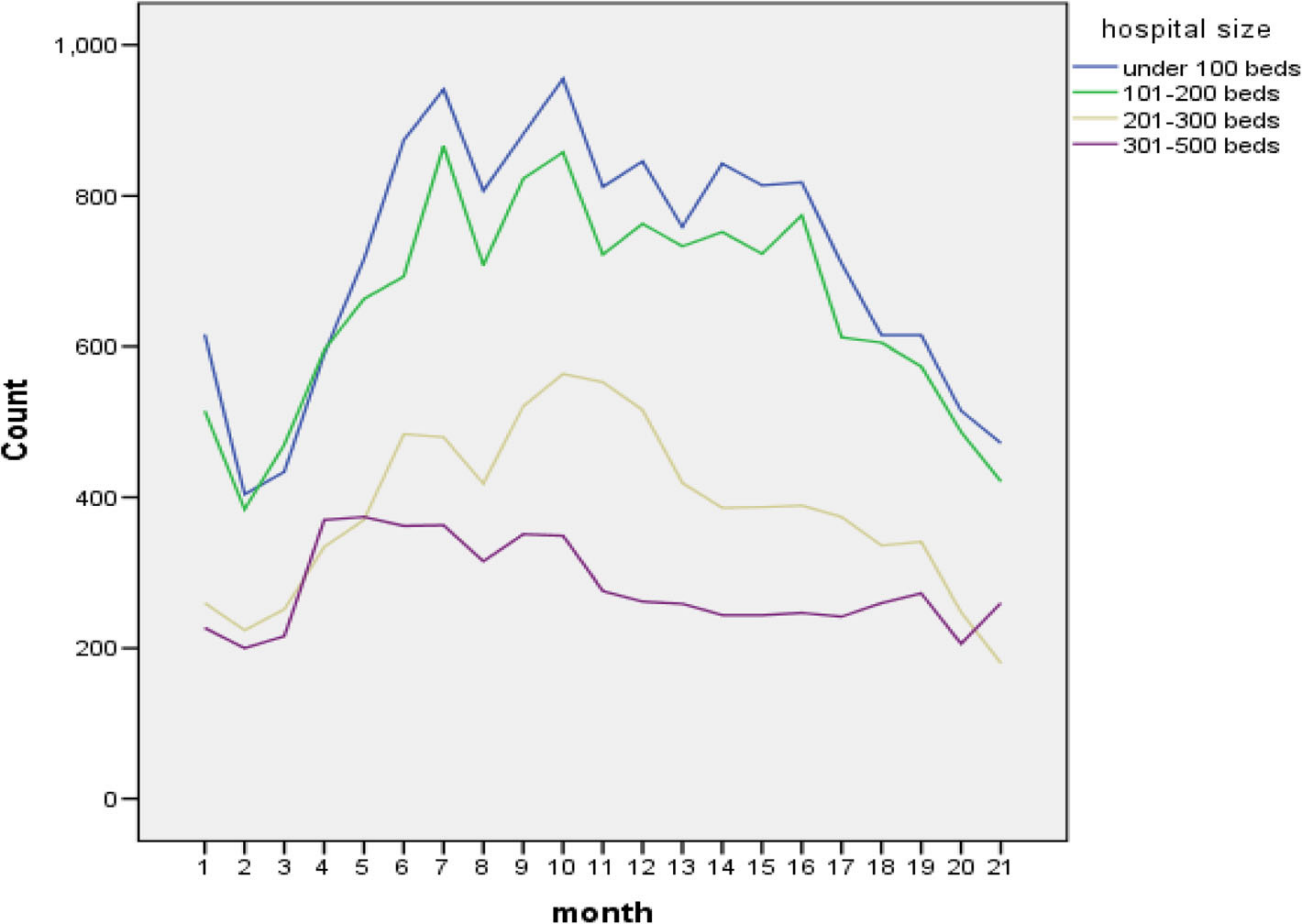
Trends of hospital claims by size over the study period

**Fig. 2:**
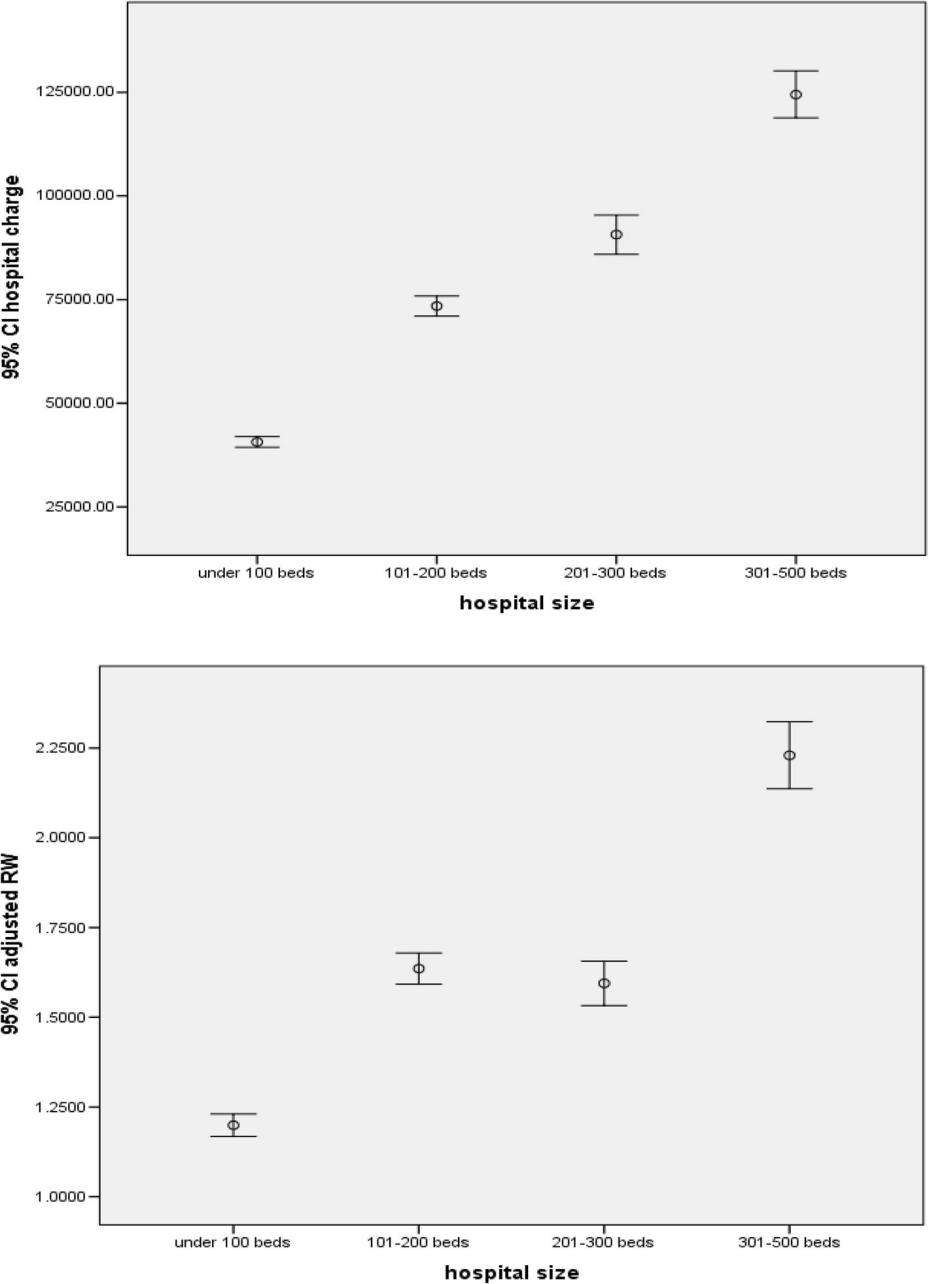
Comparison of hospital charge (upper panel) and adjusted RW (lower panel) by size

**Fig. 3:**
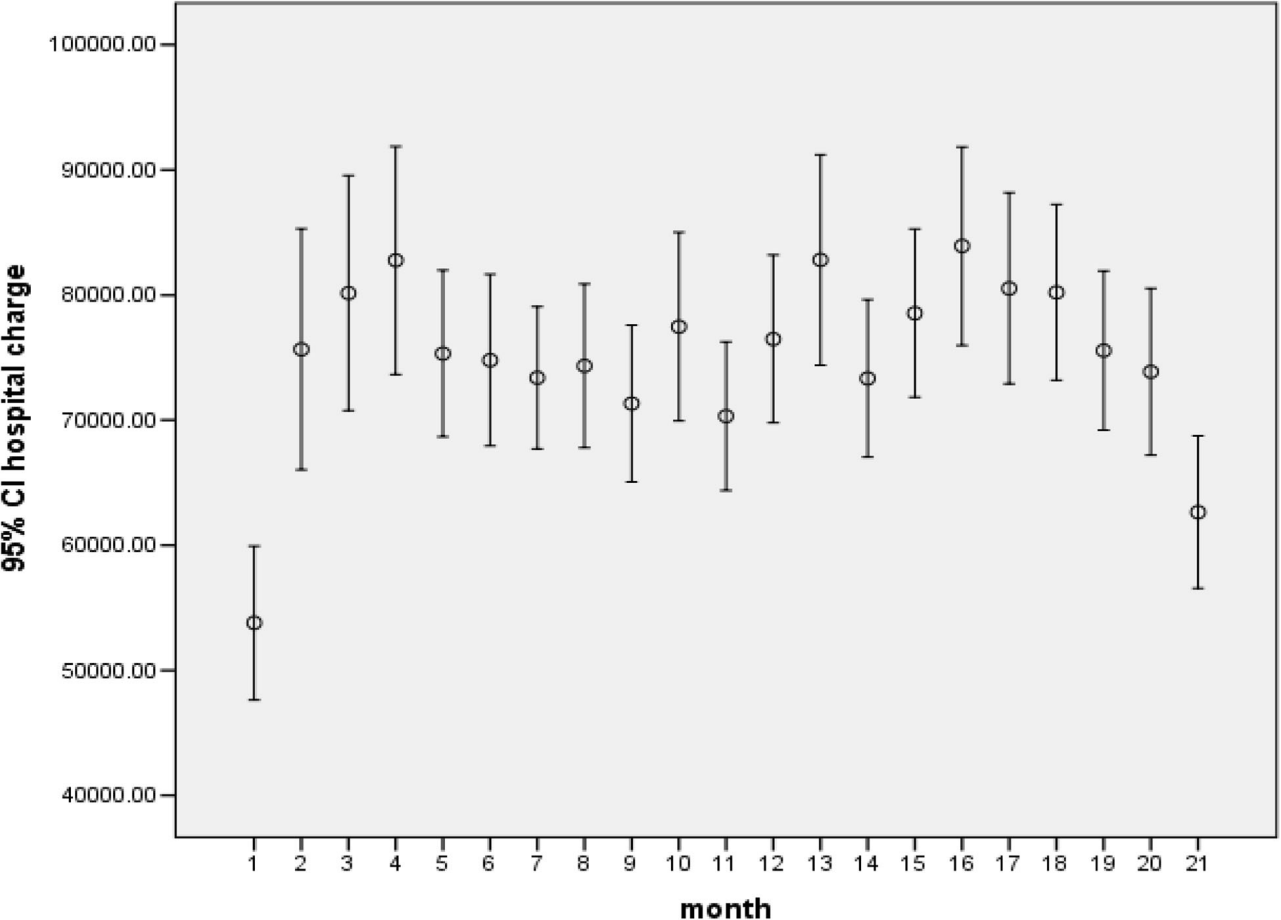
Trends of hospital charge (95 % CI of the means) over the study period

**Fig. 4:**
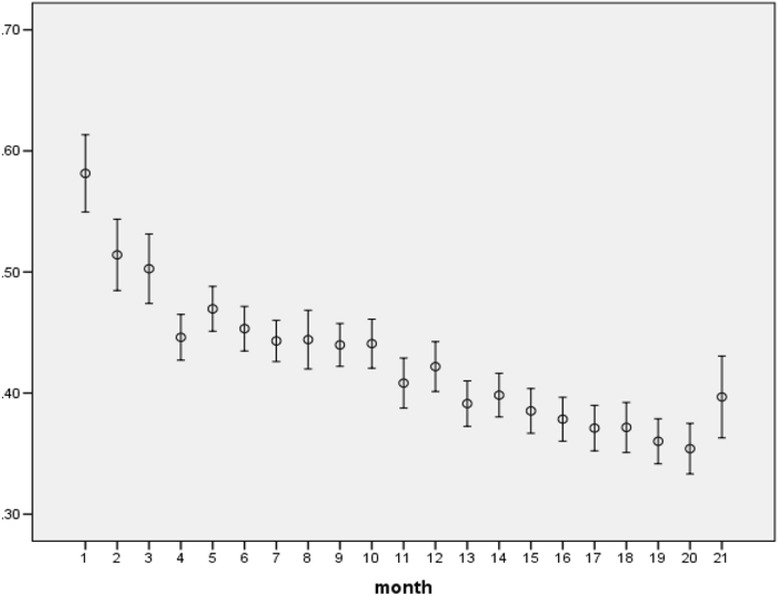
Trend of paid-charge ratio (95 % CI of the means) over 21 months (excluding the ratio = 0)

We performed statistical analyses using IBM SPSS Statistics version 18.0 (IBM Co., Armonk, NY, USA). Profile of patients in the 3 groups of health insurance schemes and monthly access to inpatient care were compared using percentage for categorical data and mean for continuous data (Table 3). We have presented monthly case mix index (CMI) and fatalities by severity over 21 months in percentages (Table 4). CMI, calculated by dividing sum of relative weights by number of patients for each month. Given heterogeneous size of the hospitals and expected differences for charging among them, it is interesting to know how financial claims from the hospitals with different size might vary over time and how hospital charges might vary among hospital of various sizes. Data are presented accordingly in Figs. 1 and 2. Then we show a plot of average amount with 95 % confidence interval (CI) of monthly claims from these hospitals over the 21 months in Fig. 3. Across the 21-month period, average paid-charge ratios with 95 % CI are presented when the figures are greater than zero (Fig. 4). This cut-off value was chosen due to substantial number of records with no payment. To explore the trends of claim processing status (pending or rejected) by month of the policy implementation, monthly approved claims were subtracted from monthly total claims then percent of claim processing status for each month was estimated as shown in Table 5. Factors related to hospital charge were investigated using multiple linear regression analysis (Table 6). Finally, we extracted findings from the Clearing House reports on a series of telephone surveys on the demand side and summarize in Table 7.

**Table 3:** Profile of patients with health insurance status, severity and monthly access to inpatient care (row%)

	CSMBS (*N* = 25,834)	SSS (*N* = 2,621)	UCS (*N* = 14,757)	Overall (*N* = 43,212)
Mean age (95 % CI)	65.2 (64.9,65.5)	40.7 (40.2,41.2)	47.3 (46.9,47.8)	
% male	45.3	50.2	56.1	
Percentage of access	59.8 %	6.1 %	34.1 %	100 %
Percentage of beneficiary in 2013^a^	8.6 %	15.4 %	74.4 %	100 %
Severity				
Critical	36.20 %	42.70 %	51.70 %	41.90 %
Urgent	57.10 %	50.20 %	45.60 %	52.70 %
Non urgent	4.10 %	4.60 %	1.50 %	3.30 %
Non-EMS	2.60 %	2.50 %	1.20 %	2.10 %
Month of policy implementation				
1	28.9 %	11.0 %	60.1 %	1,232
2	42.6 %	9.4 %	48.0 %	1,400
3	50.7 %	6.1 %	43.2 %	1,908
4	56.1 %	6.2 %	37.7 %	2,149
5	56.4 %	5.1 %	38.5 %	2,442
6	61.5 %	4.8 %	33.6 %	2,696
7	58.9 %	6.2 %	34.9 %	2,276
8	59.3 %	5.8 %	34.9 %	2,597
9	64.4 %	5.2 %	30.4 %	2,750
10	61.9 %	5.4 %	32.8 %	2,372
11	62.5 %	6.0 %	31.5 %	2,414
12	64.4 %	5.7 %	29.9 %	2,194
13	63.0 %	6.3 %	30.8 %	2,252
14	62.5 %	6.0 %	31.4 %	2,192
15	65.0 %	5.3 %	29.7 %	2,253
16	65.4 %	6.2 %	28.4 %	1,953
17	66.4 %	5.5 %	28.1 %	1,836
18	64.1 %	4.7 %	31.2 %	1,820
19	63.9 %	6.7 %	29.4 %	1,470
20	60.5 %	6.3 %	33.2 %	1,346
21	55.4 %	7.2 %	37.4 %	1,232

^a^National Statistical Office. Executive Summary. Health and welfare survey B.E. 2556 (2013) http://service.nso.go.th/nso/nsopublish/themes/files/healthy/healthyExec56.pdf [in Thai]

**Table 4:** Monthly case mix index (CMI) and fatality (%) by severity

Month of policy implementation	CMI	Critical (*N* = 18,336)	Urgent (*N* = 22,569)	non urgent (*N* = 1,387)	Monthly subtotal
1	1.59	15.1 %	0.7 %	0.0 %	1,640
2	1.93	16.3 %	1.4 %	0.0 %	1,230
3	1.91	18.2 %	1.5 %	8.3 %	1,394
4	1.87	16.9 %	1.7 %	0.0 %	1,904
5	1.77	17.1 %	1.6 %	0.0 %	2,144
6	1.63	15.8 %	1.2 %	6.7 %	2,439
7	1.69	14.1 %	1.3 %	0.0 %	2,689
8	1.54	15.9 %	1.5 %	0.0 %	2,275
9	1.54	13.9 %	1.0 %	0.0 %	2,588
10	1.59	15.7 %	1.4 %	1.8 %	2,735
11	1.44	11.1 %	1.4 %	0.0 %	2,344
12	1.55	13.4 %	2.2 %	0.5 %	2,332
13	1.57	17.7 %	2.0 %	0.5 %	2,051
14	1.47	13.1 %	2.4 %	0.5 %	2,149
15	1.48	13.1 %	2.0 %	0.0 %	2,109
16	1.49	15.3 %	1.2 %	1.3 %	2,142
17	1.49	5.9 %	0.9 %	9.1 %	1,877
18	1.48	6.9 %	2.9 %	6.7 %	1,771
19	1.52	5.8 %	4.2 %	9.1 %	1,755
20	1.34	6.2 %	1.7 %	0.0 %	1,414
21	1.03	6.8 %	2.7 %	0.0 %	1,310
overall		11.7 %	1.6 %	0.9 %	42,292

**Table 5:** Percent distribution of claims being rejected or pending by month of policy implementation

Month	Pending or rejected	Claims
	Percent	N
1	17.5	1,642
2	16.0	1,232
3	16.0	1,400
4	14.9	1,908
5	14.4	2,149
6	20.0	2,442
7	28.6	2,696
8	26.1	2,276
9	31.8	2,597
10	38.6	2,750
11	49.7	2,372
12	50.4	2,414
13	51.0	2,194
14	54.9	2,252
15	57.3	2,192
16	59.1	2,253
17	59.0	1,953
18	58.6	1,836
19	61.3	1,820
20	61.2	1,470
21	70.4	1,346

**Table 6:** Factors associated with hospital charge according to multiple linear regression analysis (*N* = 41,550; 4.7 % missing value)

Variables	Unstandardized Coefficients		Standardized Coefficients	*t*	Sig.
	B	Std. Error	Beta		
(Constant)	15464.911	4807.347		3.217	.001
Age	377.869	30.367	.063	12.443	.000
Month	463.332	142.714	.016	3.247	.001
Adjusted RW	34692.841	267.856	.631	129.521	0.000
Paid charge ratio	−97518.033	1901.460	-.250	−51.286	0.000
Severity	803.070	519.021	.008	1.547	.122
Hospital size	8239.254	733.076	.056	11.239	.000
Hospital location	1165.675	120.335	.048	9.687	.000
Sex	−1831.117	1478.853	-.006	−1.238	.216
Health insurance status	−3013.317	814.306	-.019	−3.700	.000

**Table 7:** Summary of telephone survey findings by the Clearing House

Topic	1 April-30 June 2012	1 April-31 July 2013	1 October-December 2013
Number of cases contacted (%inpatient)	673 (72 %)	640 (100 %)	560 (79 %)
Number of response (%)	351 (52 %)	461 (72 %)	321 (57 %)
EMS conditions			
Injury (%)	62 (18 %)	39 (8 %)	87 (27 %)
non-injury (%)	289 (82 %)	422 (92 %)	234 (73 %)
severity			
critical	185 (53 %)	216 (47 %)	N.A.
urgent	166 (47 %)	245 (53 %)	N.A.
copayment			
not being requested	164 (47 %)	23 (5 %)	92 (29 %)
full payment	13 (4 %)	415 (90 %)	96 (30 %)
partial payment	79 (23 %)	23 (5 %)	133 (41 %)
% missing	27 %	0 %	0 %
Patient understanding of the definition of emergency conditions			
% well understand	28	16	N.A.

*N.A*. not available

#### Qualitative data

Digital electronic records of the in-depth interviews conducted during the first three months of the program were transcribed verbatim. The key interview questions included opinions on the policy, operational definitions, mechanism and process of policy implementation, regulatory function and mechanism, provision of care and claim process, and feedback on payment system. We performed thematic content analysis to capture emerging themes. Subthemes and details are organized under the emerging themes. Relationships between themes and subthemes are identified. These were carried out in parallel by the first three authors. The authors discussed the findings to achieve consensus on the conclusions. Similarly, we applied the processes to documentary reviews. Table 8 summarizes key findings from the in-depth interviews.

**Table 8:** Summary of key findings from in-depth interviews

Emerging themes	Regulators	Private hospital directors, financial staff and ED staffs	The clearing house administrators	The administrators of the health insurance schemes
Opinions about the policy initiatives	1. The policy maker makes reference to the Sanatorium Act B.E. 2541 under the MOH to recruit the private hospitals into the policy implementation. This is perceived to be a very rare case since the law came into effect over a decade.	1. The policy makers give too short notice for private hospitals to consider whether to participate or not. 2. The single payment rate is considered inappropriate for the cost of provision of EMS which could substantially vary from case to case. 3. The rationale and method used for determining single payment rate are considered unclear. 4. Prior to the policy initiatives, the private hospitals claim of having been providing EMS to any patients in an emergency condition, even free of charge, on ethical ground and as required by the law. Hence the policy requirement is considered redundant.	1. The hectic manner in policy implementation precludes adequate design of payment rate and timely responses from the health insurance schemes in terms of modification of rules and regulations to support the policy requirement (single payment rate and payment mechanism).	1. The policy makers give too short notice for the health insurance schemes to get organize in response to the policy. The single payment rate to private hospitals and the clearing house mechanism are the issues of major concern since there is no the existing rules and regulations to support the scheme compliance to the policy requirement. 2. The UCS administrator advocates for the implemented payment rate out of a concern of financial sustainability since per head budget for the UCS is the lowest among the 3 schemes.
Operational definitions for emergency conditions (EC) and the state of being stabilized enough for inter-hospital transfer.	The definition has been used to guide prehospital care ambulance for many years. It was developed based on the U.S. standards.	1. The operational definition of EC is not clear cut hence rendering abuse from some users with non EC. There are examples of users choose to visit a private hospital far away from the scene of emergency event instead of choosing the nearby hospitals. 2. The definition for judging whether a patient is stable for inter-hospital transfer is not available. This induces difficulty in transferring the patients especially CSMBS beneficiaries to other hospitals especially public hospitals in case the patients could not afford the cost of further stay beyond 72 h in the private hospitals.	The operational definition for severity classification of EC is too subjective especially for discriminating patients with non-critical conditions. As a result, misclassification of the patients among severity categories could render difficulty in claim approval.	1. SSS beneficiaries with EC who are admitted to hospitals outside the contract are allowed to stay no more than 72 h before transferring to contracted hospitals. Hence the lack of definition on patients being stabilized for inter-hospital transfer is less likely to affect SSS beneficiaries. Similar arrangement like this does not exist in the health insurance system under CSMBS and UCS. However, the UCS beneficiaries are less likely than those of CSMBS to face difficulty in the inter-hospital transfer issue since the formers are registered to certain hospitals whereas the latters are not required to register to any hospitals.
Mechanisms and processes to enhance common understanding and acceptance of the definition among the key stakeholders	1. Formal meetings were held among all relevant stakeholders to inform detailed definition and its implications for service provision.	1. Sensible approach to the adoption of such definition should be based on consultation of providers and professional organizations such as the Royal College of Surgery etc.	1. Apart from a formal meeting to inform providers and the health insurance schemes about the definition, a public telephone number was set up to facilitate clarification of relevant concerns of stakeholders since day one of the implementation. 2. Formal training sessions were organized to assist application of the definition by the private hospitals.	1.A formal meeting was held among the administrators of the health insurance schemes at the commencement of the policy.
Regulatory function and mechanism to enhance provider compliance to the laws	1. Until the present, there has not been any formal mechanisms to keep track on the compliance of private hospitals to the Sanatorium Act. 2. So far based on my experience (a senior health officer) in working in the Health Facility Committee under the Sanatorium Act, there has not been a single case of specific private hospitals being seriously dealt with for misconduct.	Not applicable	1. Financial audit of claimed data on case by case basis is mandatory for compensation to the private hospitals. 2. Time limit has been imposed on the process of financial audit in order to avoid unnecessary delay of the compensation to the hospitals.	Not applicable
Provision of care and the process of claim submission or claim processing	1. There has not been any concrete information systems to keep track of service provision in private hospitals except for setting up online channel for user complaints.	1. Some security-market registered hospitals describe network of member hospitals with certain degree of differentiated specialization to support patients with specific needs and different level of purchasing power. The network, hence, is in a better position to smoothly handle continuity of care including inter-hospital transfer of patients with diverse needs and purchasing power. In addition, these hospitals also contend that maintaining standby teams of medical specialties for any major emergency cases is costly hence justifying the price setting. 2. A few security-market registered private hospitals show concrete evidence of standard protocols for certain EC like acute abdomen, acute chest pain. 3. It is the duty of a physician in charge of specific patients in EC to do severity classification. Charge processing is the responsibility of financial staffs taking into account the standard claim protocol of the clearing house.	1. A standard online claim protocol is specifically established for the program. According to the protocol, expected timeline for claim processing is set to be completed within a month from the date of claim submission. 2. Only financial audit is conducted to ensure hospital compliance to the protocol. 3. There is no pre-admission authorization for each hospitalized patient.	Not applicable
Feedback on the payment and the regulation	Volume of prehospital ambulance services by the national lead agency of emergency care system is reported to the high-level decision-making forum on monthly basis.	1. Without acceptable financial compensation to private hospitals, compliance to the law is hardly achievable. 2. The private hospitals proposed revision of the single payment rate to reflect the cost of service provision through participatory approach. 3. Private hospitals set prices to reflect full cost recovery and a surplus of under 15 % per annum 4. Given the fact that a number of patients bypass the hospitals closest to the place of events, it is justified to impose copayment to mitigate this misconduct on the demand side.	Monthly feedback to the high-level decision-making forum involves trends of : volume of service, access by health insurance status, number of patients by severity classification, type of hospital visits, findings from periodic telephone surveys, number of participating hospitals, duration of arrangement for inter-hospital transfer of inpatient, copayment	1. It is not clear about the progress on the attempts to make changes in rules and regulations for payment mechanism relevant to the program expectation. 2. CSMBS and SSS administrators concern about potential impacts of the expected changes on all other benefit packages for non-emergency conditions.

## Results

### Volume of services, case-mix and severity of the patients

Analysis of the EMCO dataset reveals 43,588 patient claim visits to 225 private hospitals (88.2 % of the total eligible 255 private hospitals) during the first 21 months of the program (April 2012 - December 2013). The majority of claims (91 %) were for inpatient care. Table 3 shows CSMBS beneficiaries constituted the majority of total inpatients (59.8 %) then UCS beneficiaries came second (34.1 %) and SSS beneficiaries were the smallest proportion (6.1 %). This is discordant to the percentage of total number of beneficiaries for each group. The number of inpatient claims for each month varied from 1,232 to 2,750 cases with a fluctuating trend and peaked at the 9th month. While percentage of access of CSMBS beneficiaries started at the lowest in 1st month then increased 1.8 times later on, those of UCS and SSS beneficiaries showed the opposite trends. Regarding severity among the three groups, CSMBS beneficiaries were hospitalized with the lowest percent (36 %) of critical conditions as compared to those of the others (Table 3).

The majority of patients reportedly visited with urgent conditions (22,569 cases or 53 %) shown in Table 4. Overall, those with critical conditions encountered a fatality of 11.7 % which is 10 times more than those with other conditions. However, the trends of fatality for each severity group were not stable. The last five months observed a sharp drop in fatality of those with critical conditions, roughly a third of those in earlier months. This happened in parallel with a sharp increase in the figures for those with urgent or non-urgent conditions. These findings indicated misclassification in severity of the claim records. Nonetheless, CMI seemed to follow a steadier declining trend. The percentage of cases for each 10th International Statistical Classification of Diseases and Related Health Problems (ICD-10) category with top three highest figures respectively was 21 % for the respiratory system, 15.2 % for digestive system, and 14.6 % for certain infectious and parasitic diseases.

### Hospital charge and reimbursement

Hospitals participating in the program varied in size from 10 to 500 beds with a median of 150 beds (top quartile 230 beds and bottom quartile 100 beds). Over the period, claims from small hospitals (under 200 beds, Fig. 1) dominated the whole picture. While the number of claims from big hospitals (over 300 beds) dropped continually after the 4th month, those from other hospitals seemed to follow an inverted U shape with 2 consecutive sharp drops at 10th month and the 16th month respectively. Hospital charge per case varied from 1,000 to 5.5 million baht (33.3 to 183,333.3 USD using an exchange rate of 1 USD for 30 baht) with a median of 28,018 Baht (934 USD). Average charge positively correlated with hospital size (upper panel, Fig. 2) which was similar to the correlation between adjRW and hospital size (lower panel, Fig. 2). In terms of payment, hospitals were reimbursed at a minimum of 0.0002 to a maximum of 9.9 times the total amount of charge with a median of 33.5 % of the total amount. At the top quartile, hospitals were reimbursed at 56 % of the total amount and 19.8 % at the bottom quartile. Faced with a perceived under payment, hospitals might react by upward adjustment of the charge.

On one hand, average monthly hospital charge showed a fluctuating trend over the 21 months (Fig. 3), whereas monthly CMI seemed to follow a slight downward trend (Table 4). On the other hand, paid-charge ratio followed a sharp downward trend during the same period, (Fig. 3) together with a drastic drop of the approved claim rate from 82.5 to 29.6 % during the period (overall claim approval was 59.3 %) (Table 5). These seemingly contradictory pieces of evidence from each side might indicate upward adjustment of hospital charge per case or per service item given fixed reimbursement rates. It might also indicate changing mix of hospital size with a tendency of big hospitals (over 300 beds) withdrawing from the program quite early (Fig. 1). These observations are in accordance with further analysis as follows. Over the 21 months, percentage of monthly claims being rejected or pending was steadily increasing from the first month onward (Table 5). Using multiple linear regression analysis (Table 6), it was found that the total amount of hospital charge was significantly associated with the following factors (in order of magnitude according to standardized beta coefficient): adjRW, paid charge ratio (ratio between total amount of reimbursement and total amount of hospital charge), age, hospital size, hospital location, month of hospital visits, and health insurance status except for sex and severity. This means the amount of hospital charge increased according to the value of adjRW. Big hospitals charge higher price than small hospitals (Fig. 2). Hospitals in Bangkok charge more than hospitals outside Bangkok although consumer price index for Bangkok was not the highest in the country during 2012 and 2013 [21]. The amount of charge followed an increasing trend from the beginning to the end of the 21-month period. It increased against paid-charge ratio and increased with age of the patient. The amount decreased when tracking from that for CSMBS beneficiaries to those for SSS and UCS respectively.

Up to this point, the interactions between the hospitals and the Clearing House might be better understood taking the viewpoint of complex adaptive systems (CAS) [22]. According to this viewpoint, healthcare system is considered a system which consists of elements interacting in freedom and with the ability to respond to stimuli in many different and fundamentally unpredictable ways. Adopting the CAS perspective, it follows that the hospitals play out the game by increasing charges or withdrawing (Fig. 1) based on perceived under payment and the payers (through the Clearing House) counteract by claim rejection or adherence to the payment rates. Additionally, the hospitals could also employ copayment as a strategy to cope with perceived under payment given the absence of laws or regulations precluding copayment. The next section is going to deal with this notion.

### Copayment and related factors

With the perceived under payment, hospitals might opt for copayment in addition to the upward adjustment of charges or withdrawal. Based on the paid-charge ratios, it could be inferred that the hospitals might request for a substantial copayment from roughly 40 to 80 % of the total charge. This notion is supported by evidence from the consumer surveys. As part of the feedback to the policy implementation, the Clearing House ran a series of telephone surveys in 3 consecutive periods (1st–3rd months, 7th–9th months and 12th–15th month), which showed the prevalence of copayment varied from 53 to 95 % as shown in Table 7. Results from our telephone survey (*N* = 128) for the first month of the program found the prevalence (%) of copayment varied from 80 % for CSMBS beneficiaries to 50 % for UCS and 30 % for SSS beneficiaries. The prevalence of copayment requirements reflects the fact that private hospitals perceived the reimbursement rate set by the Clearing House as under compensation to the cost of services.

The discrepancy in the amount reimbursed and the total charge clearly demonstrates a conflict between pricing policy of the hospitals and the adopted payment policy. To support the pricing policy, the hospitals argued that they set prices to reflect full cost recovery and a surplus of under 15 % per annum (Table 8). Based on this argument, we explored our previous report on pricing of the hospitals using EMCO dataset for the first 3 month period focusing on patients in critical or urgent conditions (*N* = 1,257 records). We found different prices per adjRW for the services among 3 categories of hospitals: security-market registered hospitals, general private hospitals, and not-for-profit private hospitals. On average security-market registered hospitals are bigger than the others (174 vs 81 beds per hospitals). The median price varied as follows: 47,246 Baht, 38,565 Baht and 35,727 Baht, respectively (*p*-value = 0.0001). Further analysis taking account expected differences in fixed and operating cost between hospitals in Bangkok and regional provinces, we found no difference in price among security-market registered hospitals operating in Bangkok or regional provinces. To the contrary, the differences were demonstrated for the other two categories i.e., higher price for hospitals operating in Bangkok and lower price for hospitals in regional provinces. These findings vividly indicate different pricing policies among different categories of the hospitals. It should be noted that security-market registered hospitals charged the highest price despite the fact that they are in a better position to access loans at the lowest interest rates compared to the others. Finally, in the same report, our team conducted a head-to-head comparison of itemized prices (medications and medical supply) on 80 selected patients of the private hospitals to those prices of a tertiary care public hospital to shed light on rational of the pricing. We found that medication prices differed from 1.15 to 399 times and 1.55 to 4.4 times for medical supply. By reviewing medical records and hospital bills, we also found seemingly unnecessary charge items on the hospital bills. For instance, the items included a screening test for prostate cancer in a patient with pneumonia, bone fixation in a patient with hypertension, and blood transfusion without proper medical indications. Results of the head-to-head comparison and the review of medical records cum hospital bills suggested inappropriate pricing of some private hospitals. After viewing these findings, reaction from representatives of the hospitals turned out quite predictable. They argued that private hospitals have to fully rely on themselves from the first to the last dollar in capital investment and daily operation, which is not the case for public hospitals. As a result, they argued comparisons are not appropriate. With regard to the findings from the review of medical records and hospital bills, they did not give any opinion.

In-depth interview provided further evidence related to the copayment level. Private hospitals cited unclear operational definitions of emergency conditions as a reason to impose copayment to deter perceived abuse of the services by patients. This perception emerges from the hospitals report of encountering patients with non-urgent conditions requesting free access to ED and inpatient care. This notion sounds logical given the large proportion (90 %) of patients accessing ED by informal means [15]. This renders the prehospital care triage systems (with standard criteria of severity classification) irrelevant. Next, the hospitals also reported patients bypassing closest public hospitals to their facilities. This finding corresponded to the finding from our telephone surveys (*N* = 128) revealing the most common reasons for bypassing closest public or private hospitals were due to a perception of inferior quality of care. Finally, the private hospitals vindicate copayment by attributing to the lack of criteria to justify inter-hospital transfer after the first 72 h of admission while the patients demand further stay.

### Patient’s voices and policy communication

Our telephone survey on the 128 patient samples revealed over 90 % of them were ignorant of the channel for making complaint to the Clearing House. During the early months of the program, analysis of complaint calls from the patients or relatives to the Clearing House identified 1,165 calls asking for clarification of eligible emergency conditions; 633 calls of eligible hospitals for access; and 409 calls of the conditions for copayment. These findings indicate inadequate public policy communication to the users.

### Public governance of the hospitals and the public financial arrangement

The high degree of freedom in price setting by the hospitals raises an issue of hospital regulatory mechanism. The Sanatorium Act B.E. 2541 (section 32) requires hospitals to disclose prices of medical and hospital services which hospitals have to comply with accordingly. To enforce the laws, the Health Facility Committee has been set up and chaired by the Permanent Secretary of the MOPH. Its voting members consist of the Heads of 4 departments of the MOPH, a representative of the Council of State and of the Office of Consumer Protection. In addition, the committee includes 3 health practitioners, a representative of each of the health professional councils and technical advisors which have to be representatives from the private hospitals for at least 1 person and no more than 3. The Committee has been tasked with issuing regulations, granting permission for establishing and operating a hospital, promoting quality improvement and processing appeals. Since the law has been in place for almost two decades, there have not been any information systems to keep track of the hospital behaviors. Alarmingly, informal interview found a key informant, (a senior health officer with direct experience in working on the committee) who maintained that he had never witnessed a single case of hospitals being seriously dealt with for misconduct. This notion is in keeping with findings from the review of the annual reports in 2006–2014 of the department overseeing the Sanatorium Act, which found no evidence pertinent to the law enforcement [23].

Turning to the payment rate issue, in-depth interview with high-ranking officers of the Clearing House and the health insurance schemes revealed the rationale behind the DRG-based payment rate. We find the rate was being set without concrete evidence about the cost of hospital services except for a concern of future financial burden to the UCS if a higher rate than the current one would have been adopted. However, in effect, a higher rate of DRG-based payment to private hospitals has been adopted for other previous programs under the UCS for medical services for premature newborns, patients with severe head injury, cardiac catherization etcetera.

Finally, in-depth interview of the administrators of the 3 schemes revealed no concrete evidence of any progress in the attempts to make changes in rules and regulations pertinent to the program requirement i.e. harmonizing the payment mechanism and payment rates to facilitate access to EMS of the hospitals. Hence, the existing payment made possible using the financial resources and personnel of the UCS assigned to take major responsibility in the program execution through the Clearing House. Major obstacles cited by the administrators of SSS and CSMBS are the concern of potential impacts of the expected amendment of rules and regulations on all other benefit packages for non-emergency conditions.

## Discussion

### Copayment and its effects

Using a mixed methods, this report is able to shed light on the existence of copayment despite the public financing of access to the hospital provision of EMS during the 21 months of the program implementation. The copayment existed in parallel with inequitable access to EMS among the beneficiaries of the 3 schemes as shown in Table 3. A previous survey of 3,504 ED users in public hospitals in the 4 regions of the country revealed different percentage of access among the beneficiaries in contrast to the figures. in Table 3 i.e., 10.2 % for CSMBS, 6.0 % for SSS and 68.5 % for UCS [24] Similar figures from an earlier ED survey of 6,440 patients was also documented [12]. The copayment also coexisted with inequitable health outcomes as reported in a previous study based on analysis of the EMCO dataset [15]. Evidence from developed countries on the effects of copayment on access to hospital ED or other forms of healthcare, nonetheless, has been inconclusive as mentioned in the introduction. A recent report using difference-in-difference analysis of data over a 10-year period did not demonstrate a reduction in ED use following the enforcement of copayment in the U.S. [20] The authors ascribe this negative finding to several factors. First, difficulty in collecting the copayment out of fear of violating the provisions of the Emergency Medical Treatment & Labor (EMTALA) Act Law, which requires hospitals to provide appropriate medical screening to persons seeking medical care through an ED, and that hospitals treat and stabilize anyone with an emergency medical condition. Second, ED health care providers may have difficulty determining whether a visit was due to a medical emergency or was non-urgent. There has been no consensus on what constitutes a non-urgent visit, which helps to account for the wide variation in estimates for non-urgent ED visits (range, 8–60 % of all ED visits). Third, ED staff are required to give the beneficiary the name of an accessible Medicaid health care provider, which, given the national shortage of Medicaid health care providers, may be difficult for many EDs.

### Bypassing the local gatekeepers and contracted referral hospitals

In similar to several countries in Organisation for Economic Co-operation and Development (OECD) with UHC, under the policy directive Thai patients in urgent conditions are allowed to directly seek hospital ED without copayment [25]. However, the policy put emphasis on seeking ED closest to the place of emergency events. To the contrary, our findings documented hospitals concerned about bypassing issue and used this issue to justify copayment (Table 8). Viewing from the demand side perspective, our telephone surveys found perception of inferior quality of care as main reason for bypassing. In the Netherlands, Valk et al. found expectation of diagnostic tests not available at general practice clinics as the main reason for patients bypassing to hospital ED [26]. The next common reason was patient being already under specialist care at the hospital. More or less, these findings could be considered in the same vein as our report of the patients’ perception of inferior quality of care. Perhaps, these reasons may influence some countries (such as England and Denmark) with strong gatekeeping mechanisms in place to introduce new initiatives designed to facilitate access to specialists [25].

The declining percentage of patients with critical conditions over the 21-month period shown in Table 4 may reflect a demand for after-hour specialist care. Schoen et al. reported difficulty in getting care nights, weekends, or holidays was of significant concern among adults in all five OECD countries (Canadian and U.S. adults especially the elderly were also more likely to have gone to hospital ED for care that their regular source could have provided if available [27]. This finding is in keeping with our results shown in Table 3 that CSMBS beneficiaries not only constituted the highest percent of ED users but also were the oldest among all groups of beneficiaries. Taking together the findings of OECD countries and ours, ED could be deemed a sensitive indicator for how healthcare systems are responding to patients’ needs. And in response to after-hour healthcare needs, those OECD countries came up with different initiatives such as after-hours general practitioner clinics co-located with ED in Australia, a Primary Health Care Transition Fund to encourage models of round-the-clock, team-based care in Canada, and twenty-four-hour nurse-staffed hotline and NHS Walk-In Centres in the United Kingdom [27]. Similarly, many public hospitals in Thailand currently provide after-hour clinics from 5 to 8 pm on weekdays and 9–12 am on Saturday. Nonetheless, there has not been any systematic assessment as to the impacts of these after-hour clinics in meeting the demand using ED as an indicator.

### Strategies for dealing with copayment

These difficulties confronting the U.S. hospitals (mainly private) are, in turn, instructive to improvement of the provisions and enforcement of the Sanatorium Act in Thailand. First the Sanatorium Act should include provisions with more specific details requiring hospitals to provide screening, stabilizing and transfer for the patients similar to those of EMTALA. Provisions for penalty should also be clear and substantial enough like that of EMTALA. Apart from the provisions; the effectiveness of enforcing EMTALA lies on the so-called “Medicare death penalty” i.e. termination of Medicare contract to hospitals based on result of investigation of potential law violations by the Centers for Medicare and Medicaid Services (CMS) [28]. The death penalty is meaningful to the United States hospitals because of arguably substantial financial contribution from CMS to the hospitals. This is not quite the case in Thai context in which major revenues for many private hospitals come from private insurance and OOP. In addition, with diverse payment rates and methods among the 3 schemes functioning independently, it is difficult to impose meaningful financial sanctions to the private hospitals. In this regard, harmonization of payment methods and rates of the 3 schemes should take into account strengthening the bargaining power of public healthcare financing against the private hospitals. Finally, enforcement of the Sanatorium Act would need to put in place an investigative mechanism like that of CMS. This involves impartial status of the investigators and sufficient financial support for the investigation.

Concerning well-informed decision of the users, the findings on limited knowledge of the users indicate inadequate policy communication based mainly on mass media and out-door media. This could be addressed through regulatory measure mandating the hospitals to make formal notification of the right of access to every patient visiting ED as is the case under EMTALA. In many European countries, hospitals are required to bear the burden of proof in case of liability for malpractice and errors in providing information [29]. This kind of legal provision could enhance the sensitivity of the hospitals to users’ need for relevant information.

Review of national efforts among BRICS countries (Brazil, Russia, India, China, and South Africa) reveals substantial contribution of private providers in healthcare provision [30]. In fact, this situation is not unique to BRICS countries but is similar to those in many other low-middle income countries including Thailand. This undeniable role of the private sector poses a specific challenge in the public sector i.e., stewarding mixed private and public health systems. Our report suggests intermingled role of stewardship (the role of regulator) and financing (the role of the three major schemes) in promoting more access to EMS in Thailand - a country with over a decade of experience in sustaining UHC. Although we are able to shed light on provision of EMS among a selected group of private hospitals not previously included in the purchasing models of the three schemes, regrettably so far there has not been any evidence on the provision of EMS by contracted private hospitals under SSS and UCS. This indicates room for improvement of relevant information systems to keep track of the service provision.

### Needs for further studies

Considering a need for strategic purchasing from powerful private sector in pursuit of UHC, the present report indicates a number of inter-related key specific issues for further studies: capacity building for healthcare financing agencies in negotiating with private providers based on rational and evidence-based price setting; and design of technically strong and high commitment of regulatory mechanism to set up effective legal framework to ensure sufficient access and fair pricing. Both these issues should be conceived along with the development of sufficient information systems to keep track of private providers in terms of service provision (access and quality) and pricing. Finally, further studies should focus on assessment of implementation of the suggested legal measures to enhance providers’ sensitivity to users’ need for relevant information at the point of care. Finally, systematic assessment on the impacts of the provision of after-hour clinics by public hospitals should be conducted taking account ED as an indicator.

### Limitations

We chose to focus on inpatient dataset hence rendering inadequate exploration of ED visits. The results of user survey could be biased due to substantial number of non-respondents or failure to make contact. We could not explore methodological details of the telephone survey of the Clearing House, which might be subject to selection bias and information bias. Without access to minutes of the executive meetings during the policy implementation, we were not able to explore interactions among policymakers and the key stakeholders. In-depth interviews were conducted during the first few months of the program, hence the findings may not be up-to-date given the dynamicity of the policy context resulting in changing faces of policymakers during the 21-month period. Finally, administration of the EMCO dataset is not sophisticated enough, hence quite substantial relevant data are missing for some variables. This deficit in data quality might affect parameter estimation.

## Conclusion

Using a mixed-methods approach to draw evidence from multiple sources, we have been able to shed light on the existence of copayment in the Thai healthcare system despite the presence of UHC since 2001. The copayment poses a barrier of access to emergency care delivered by private hospitals according to the policy proclaiming free access. The copayment differentially affects different beneficiaries of the major 3 public-health insurance schemes hence inducing inequity of access. We have identified 6 drivers of the copayment i.e., perceived under payment, unclear operational definitions of emergency conditions or lack of criteria to justify inter-hospital transfer after the first 72 h of admission, limited understanding by the service users of the policy-directed benefits, weak regulatory mechanism as indicated by lack of information systems to trace private provider’s practices, and ineffective arrangements for inter-hospital transfer. With demand-side perspectives, we addressed the reasons for bypassing gatekeepers or assigned local hospitals. These are the perception of inferior quality of care and age-related tendency to use ED, which indicate a deficit in the current healthcare systems under UHC. Finally, we have discussed strategies to address these potential drivers of copayment and needs for further studies.

## Additional files

Additional file 1: Interview Question Guideline. (XLSX 23 kb)
Additional file 2: Patient’s Interview Form. (DOCX 25 kb)

## Abbreviations

AdjRW: Adjusted relative weight
BRICS: Brazil, Russia, India, China, and South Africa
CAS: Complex adaptive systems
CMI: Case mix index
CMS: Centers for Medicare and Medicaid Services
CSMBS: Civil Servant Benefit Scheme
DRG: Diagnosis-related-group
ED: Emergency department
EMCO: Emergency Medical Claim Online
EMS: Emergency Medical Services
EMTALA: Emergency Medical Treatment & Labor
FFS: Fee for service
ID: Identification
MOPH: Ministry of Public Health
NHSO: National Health Security Office
OECD: Organisation for Economic Co-operation and Development
OOP: Out-of-pocket payments
RW: Relative weight
SSS: Social Security Scheme
UCS: Universal Health Coverage Scheme
UHC: universal health coverage
USD: United States Dollar
