# Supplementary material for: Copayment and recommended strategies to mitigate its impacts on access to emergency medical services under universal health coverage: a case study from Thailand
Source: BMC Health Serv Res. 2016 Oct 21;16:606. doi: 10.1186/s12913-016-1847-y (PMC5073698; doi:10.1186/s12913-016-1847-y)
Supplement: Additional file 2: — Patient’s Interview Form. (DOCX 25 kb) [file 12913_2016_1847_MOESM2_ESM.docx]

**[__][__][__]**

**Patient’s Interview Form**

**Part 1 General information**

**Please marks ✓ in ❒ or fills in the blanks which is the most suitable answer**

| 1. Informant 1 Patient/Hospital user   2 Son/Daughter 3❒ Parents 4❒ Spouses 5❒ Relations  Please specify reason(s) for being proxy respondents for the patient ……………………………………… | Interview  Why |
| --- | --- |
| 1. Have you ever used any service of this hospital before?   1 Yes 2 No | Member |
| 1. In this visit, was this hospital the first hospital that you go?   1 Yes 2 No  Because....................................................... | First_hos  Why_1 |

**Part 2 Interview form for patient underwent service as policy of “Medical Emergency Care for Everybody Everywhere”**

**Backgrounds**

| 1. Have you known/received information about policy of “Medical Emergency Care for Everybody Everywhere”?   1 No (go to question no.6.) 2 Yes | Know |
| --- | --- |
| 1. Which source of Emergency care policy information did you get? (able to choose more than 1 answer)   1 Hospitals announcement e.g. posters, etc.  2 Newspapers  3 Televisions  4 Billboards/Posters in other places  5 Doctors who give treatment  6 Relatives or friends  7 1669 hotline’s staff/ Medicare/ Rescue units  8 Staffs from hospital where respondent received treatment | How |
| 1. What do you define of ‘emergency’? (able to choose more than 1 answer)   1❒ state that poses immediately/suddenly need for medical attention  2❒ state that threatening the life  3❒ state that threatening the life and/or disability if patients don’t received the urgent treatment  4❒ Accidents  5❒ Any symptoms and situations  6❒ Others  Please specify…………………………………………………… | Knowledge  Other |

**Part 3 Information for access to care**

| 1. What was the illness/symptom on this hospital visit?   1 Wound/Injury  2 Traffic accidents  3 Symptoms that treated by medicine (chest pain, unconsciousness)  4 Symptoms that treated by obstetrics and Gynecology (childbirth, etc.) | Symptom |
| --- | --- |
| 1. How did you get to hospital?   1❒ Myself  2❒ Car from paramedic  3❒ Car from paramedic or ambulance car that connected to 1669/1646  4❒ Hospital ambulance via directly calling to hospital | Come |
| 1. Have you known emergency medical care hotline of 1669 or 1646 before (in case of medical emergency happening in Bangkok)?   1❒ Yes  2❒ No | Emergen_no. |
| 1. Did you need ambulance?   1❒ Yes 2❒ No | Need |
| 1. Did you know the nearest hospital to the accident scene?   1❒ No  2❒ Yes  The distance between accident scene and the nearest hospital is about ……... kilometers. | Hosp_near  Km. |
| 1. Did you decide to choose the hospital by yourself?   1❒ Yes 2❒ No | Choose |
| 1. Did the hospital that you visit is the nearest hospital of the scene of accident?   1 Yes (Skip question no.15) 2 No | Hosp_sence |
| 1. What were the main reasons that you choose this hospital? (able to choose more than 1 answer)   1❒ It is the nearest point from the accident scene  1❒ I were confident in its quality and standard of treatments  1❒ I could receive the fast service, no waiting time  1❒ Easily claim for emergency medical service insurance  1❒ The hospital fee was not expensive  1❒ Other(s) …………………………………………………………………… | Why_used |
| 1. What were the main reasons that you didn’t choose the nearest hospital for treatment? (able to choose more than 1 answer)   1❒ I weren’t confident in the quality and standard of treatments  1❒ Long waiting time  1❒ I weren’t satisfied with the services of staffs in that hospital  1❒ I concerned about expenditure  1 I didn’t choose the hospital by myself.  1❒ Other(s) | Reason |
| 1. Before visiting this hospital, would you expect to get treatment before asking your health insurance scheme?   1❒ Yes 2❒ No | Expect |
| 1. When arriving the hospital, were you asked about your health insurance scheme before treatment?   1❒ Yes 2❒ No | Claim |
| 1. In this hospital visit, which healthcare insurance did you use?   1 EMCO 2 Out of pocket (go to question no.22)  3 Other (s) please specify......................................... |  |
| 1. Have you known you have the right to get the basic emergency or life-saving treatments without asking about your health insurance scheme?   1❒ Yes 2❒ No | Care |
| 1. Have you known before arriving the hospital that you would receive the basic treatment to improve your condition, and then you would been referred to your registered hospital for further treatment?   1❒ Yes 2❒ No | Refer |
| 1. Did you accept that you would have been referred to your registered hospital of you for treatment after you received a prompt emergency treatment by the first hospital?   1❒ Yes 2❒ No | Accept_refer |
| 1. Did you pay for treatment in this visit?   1❒ No (Go to question no.25)  2❒ Yes  Amount …………………………… Baht | Pay  How much |
| 1. Did you get the reimbursement as money was paid for treatment?   1❒ Yes 2❒ No | Refund |
| 1. Was the treatment charge burden your or your family finance?   1❒ Yes 2❒ No | Burden |
| 1. Do you think that the approval process of “Medical Emergency Care for Everybody Everywhere” for health insurance payment was uncomplicated and fast?   1❒ Yes 2❒ No 3❒ Didn’t use it | Approve |
| 1. Do you known the telephone number for complaining the hospital services?   1❒Don’t know, not sure, no answer  2❒Yes, please specify................................... | No.  What_no. |
| 1. Did you satisfy with the hospital service?   1 Yes  2 No  Please specify ......................................................................... | Feel  Feel1 |
| 1. Will you recommend relatives or friends to this hospital?   1 Yes, I will  2 No, I will not. | Introduce |

**Part 4 Individual information**

| 1. How much is your monthly income?   1 No income  2 less than 10,000  3 10,001 to 30,000 Baht  4 30,001 to 50,000 Baht  5 50,001 to 70,000 Baht  6 70,001 to 100,000 Baht  7 more than 100,000 Baht | earnings |
| --- | --- |
